# Host-Range Dynamics of *Cochliobolus lunatus*: From a Biocontrol Agent to a Severe Environmental Threat

**DOI:** 10.1155/2014/378372

**Published:** 2014-06-02

**Authors:** Bengyella Louis, Sayanika Devi Waikhom, Pranab Roy, Pardeep Kumar Bhardwaj, Chandradev K. Sharma, Mohendro Wakambam Singh, Narayan Chandra Talukdar

**Affiliations:** ^1^Institute of Bioresources and Sustainable Development (IBSD), Takyelpat, Imphal, Manipur 795001, India; ^2^Department of Biotechnology, The University of Burdwan, Golapbag More, West Bengal 713104, India; ^3^Department of Biochemistry, University of Yaoundé I, BP 812, Yaoundé, Cameroon; ^4^Department of Biotechnology, Haldia Institute of Technology, Haldia, West Bengal 721657, India; ^5^Regional Centre of Institute of Bioresources and Sustainable Development (RCIBSD), Gangtok, Sikkim 737102, India

## Abstract

We undertook an investigation to advance understanding of the host-range dynamics and biocontrol implications of *Cochliobolus lunatus* in the past decade. Potato (*Solanum tuberosum* L) farms were routinely surveyed for brown-to-black leaf spot disease caused by *C. lunatus*. A biphasic gene data set was assembled and databases were mined for reported hosts of *C. lunatus* in the last decade. The placement of five virulent strains of *C. lunatus* causing foliar necrosis of potato was studied with microscopic and phylogenetic tools. Analysis of morphology showed intraspecific variations in stromatic tissues among the virulent strains causing foliar necrosis of potato. A maximum likelihood inference based on *GPDH* locus separated *C. lunatus* strains into subclusters and revealed the emergence of unclustered strains. The evolving nutritional requirement of *C. lunatus* in the last decade is exhibited by the invasion of vertebrates, invertebrates, dicots, and monocots. Our results contribute towards a better understanding of the host-range dynamics of *C. lunatus* and provide useful implications on the threat posed to the environment when *C. lunatus* is used as a mycoherbicide.

## 1. Introduction

Race specific* Cochliobolus* species have caused plant disease disaster such as the southern leaf corn blight epidemic of 1970s in the United States of America [1], northern leaf corn blight (*Exserohilum turcicum*) and corn head smut (*Sporisorium reilianum*) in northern China in the 1990s [2, 3], and the Great Bengal rice famine of India in 1940s [4, 5]. In the Great Bengal rice famine, more than 2 million people starved to death due to reduction in rice yield of about 40 to 90% [5].* Cochliobolus* species often cause diseases to several plant families including Alliaceae, Anacardiaceae, Araceae, Euphorbiaceae, Fabaceae, Malvaceae, Rutaceae, Zingiberaceae and Solanaceae [6].

*Cochliobolus lunatus* [7] and related species are extensively used as mycoherbicides for controlling weeds in paddies [8–13]. The host-range of* C. lunatus* includes plant species, namely,* Cynodon* sp.,* Oryza* sp.,* Pennisetum* sp.,* Saccharum* sp.,* Sorghum* sp.,* Triticum* sp., and* Zea* sp. [14]. Geographically,* C. lunatus* was suggested to be located mainly in Australia, Brazil, Guinea, India, Cameroon, Columbia, Ecuador, Fiji, Gambia, Guadalcanal, Malaysia, Nigeria, Pakistan, Papua New Guinea, Sierra Leone, Sri Lanka, Sudan, Tanzania, Thailand, and USA [14] but not in Europe (http://www.tifton.uga.edu/fat/disfunt2.htm). The proposed geographical circumscription and putative hosts of* C. lunatus* have not been updated.

*C. lunatus* has emerged in the last decade as a virulent and destructive pathogen [15, 16]. Remarkably,* C. lunatus* successfully thrives on important crops such as rice (*Oryza sativa* L.), wheat (*Triticum aestivum*), cassava (*Manihot esculenta*), sorghum (*Sorghum bicolor*),* Hymenaches* species, strawberry (*Fragaria* ×* ananassa*),* Amaranthus* species, and potato [16–20]. Decades after Sivanesan's [14] pioneering study, is* C. lunatus* solely endemic to the outlined geographical locations? If no, has* C. lunatus* gained hosts and new geographical zones in the last decade? The aims of this study were (1) to determine the interrelatedness of 5 virulent strains of* C. lunatus* causing foliar necrosis of potato using morphological descriptors coupled with phylogenetic tools and (2) to establish the current host-range diversity of* C. lunatus* in the last decade.

## 2. Materials and Methods

### 2.1. Study Area and Sampling

Routine survey was performed in potato plantations of Burdwan District (23°14′N, 87°51′E, altitude 150 m, 102.1 km from Kolkata), West Bengal, India, during the winter months of December to March of 2010, 2011, and 2012. Mainly potato cv. Kufri Jyoti is farmed in Burdwan District. The area receives an average annual rainfall of 1173–1442 mm and temperature of 10–20°C during potato farming season. Potato plants showing brown-to-black leaf spot disease previously described [20] were used. Brown-to-black leaf spots were excised and treated with 2% NaClO solution for 2 min and rinsed in sterile water with three changes. The leaf pieces were aseptically plated on V8 agar medium (HiMedia, Mumbai, India) and incubated at 25°C in dark. Developed colonies after 7 days were morphologically identified based on standard monograph taxonomic keys [7].

### 2.2. Host-Range Diversity

The genomic DNA was isolated from fungal isolates grown in potato dextrose broth (PDB) (HiMedia, Mumbai, India). Approximately 100 mg of mycelia mat was disrupted in the presence of TRI-reagent (Sigma, St. Louis, MO, USA) using mortar and pestle containing 2 mg/mL proteinase K (Merck, Bangalore, India) following the manufacturer instructions. The quality and quantity of the DNA were determined using a 1% agarose gel electrophoresis and a nanodrop spectrophotometer (BioSpec-nano, Shimadzu, Japan), respectively. For molecular identification, the partial sequence of 5.8S rDNA, complete internal transcribed spacer 2 region (ITS2), and partial 28S rDNA region were amplified as previously described [23]. To distinguish the strains, we designed specific primers (forward: 5′-cgatatgcggcatatgca-3′; reverse: 5′-acctacgcattgcggaa-3′) for* glyceraldehyde-3-phosphate dehydrogenase (GPDH)* gene using* C. lunatus* (GenBank accession number Gb X58718) sequence. Amplification of* GPDH* was performed as follows. The PCR mix contained 11 ng genomic DNA, 5 *μ*L Green GoTaq reaction buffer (Promega, Madison, WI, USA), 0.2 mM each of deoxyribonucleoside triphosphate (dNTP), 0.2 *μ*M of each primer, and 1.1 U of GoTaq DNA polymerase in a total reaction volume of 25 *μ*L in triplicates (PCR conditions: 5 min at 95°C, 35 cycles of 1 min at 94°C, 1 min annealing at 53°C, 2 min for extension at 72°C, and a final 5 min extension at 72°C). The quality of the amplicon was checked by performing agarose gel electrophoresis. The PCR products were purified and sequenced. Sequences were assigned to molecular species based on 98–100% sequence similarity threshold in the GenBank with the following accession numbers: JX512810, JX512809, JX907827, JX477595, and JX907828, respectively, for rDNA.* GPDH* sequences have been submitted in DNA Data Bank of Japan (DDBJ) as accessions AB859034, AB859035, AB859036, AB859037, and AB859038, respectively.

Using GenBank BLAST search tool, a studied set of rDNA sequences deposited in the last decade was collected based on the information associated with the sequences such as GC content, length (>250 bp), and geographic origin of host. Importantly, records with 100% sequence similarity from the same host and geographical coordinates were removed. Unique sequence sets were screened using ElimDupes (available at http://hcv.lanl.gov/content/sequence/ELIMDUPES/elimdupes.html). Sequence alignment was performed using Muscle program [24]. Best substitution model parameters were determined based on Akaike information criterion, corrected (AICc) and Bayesian information criterion (BIC). The evolutionary history was inferred using the maximum likelihood (ML) method, and rooting was performed automatically by saving the generated ML tree in standard Newick format and all the analysis were performed in MEGA 6.06 (updated v. 6140226) software [25]. The strength of the internal branches of the ML tree was statistically tested by performing 1000 bootstrap replications.

## 3. Results and Discussion

### 3.1. Identification of* C. lunatus* Strains Causing Foliar Necrosis of Potato

**Figure 1:**
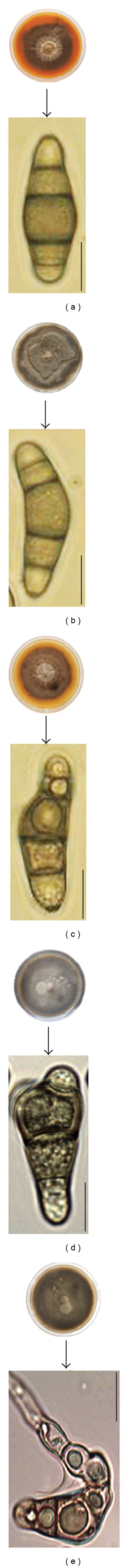
Light microscopic images showing morphological variations of five strains of* Cochliobolus lunatus* causing brown-to-black leaf spot disease of potato. (a) Strain Btl26IBSD (DDBJ accession AB859034) with no stromata, (b) strain Btl27IBSD (DDBJ accession AB859035) with no stromata, (c) Btl28IBSD (DDBJ accession AB859036) having stromatic tissue, (d) strain Btl29IBSD (DDBJ accession AB859037) having stromatic tissue, and (e) strain Btl30IBSD (DDBJ accession AB859038) having stromatic tissue. Images were acquired with Olympus DP70 camera (Olympus BX61, USA) at 1000X magnification and scale bars represent 10 *μ*m.

Basically, most* Cochliobolus* species have curved conidia, a broad rounded apex cell, a distinct swollen central cell, a tapering to narrowly round base cell, and 4-5 distinct septa. The five strains of* Cochliobolus* causing brown-to-black leaf spot disease of potato produced varied colonies and conidia (Figure 1) similar to previous studies [6, 7]. The isolates visibly produced different growth patterns (Figure 1). In one isolate Btl26IBSD (DDBJ accession AB859034), brown to whitish mycelium, reddish brown medium, and canoe five-celled conidia without stromatic tissues were observed (Figure 1(a)). In* C. lunatus*, the stromata are oval or ellipsoidal, 10 to 40 *μ*m in diameter, and located beneath the ascomata. Another isolate Btl27IBSD (DDBJ accession AB859035) produced greyish-brown mycelium and cylindrical clavated fived-celled conidia void of stromatic tissue (Figure 1(b)). Isolate Btl28IBSD (DDBJ accession AB859036) profusely produced yellowish pigmented five-celled conidia, with stromatic tissue, variable shapes, and end at one cell with a thin hilium (Figure 1(c)). Isolates Btl29IBSD (DDBJ accession AB859037) and Btl30IBSD (DDBJ accession AB859038) produced greyish-brown cottony mycelium (Figures 1(d) and 1(e)). Noteworthy, isolate Btl30IBSD profusely produced dark pigments, and with each cell of the conidia bearing a distinctive oval stromata of different sizes. The exact role of stromata in pathogenicity is not known. The stromata are enclosed by a ring of melanin-like pigment, may play a role in preventing desiccation of the conidia, conserved the gene-pool, and ensure survival under adverse conditions. As shown (Figure 1), morphological characters revealed significant intraspecific variations.

**Figure 2:**
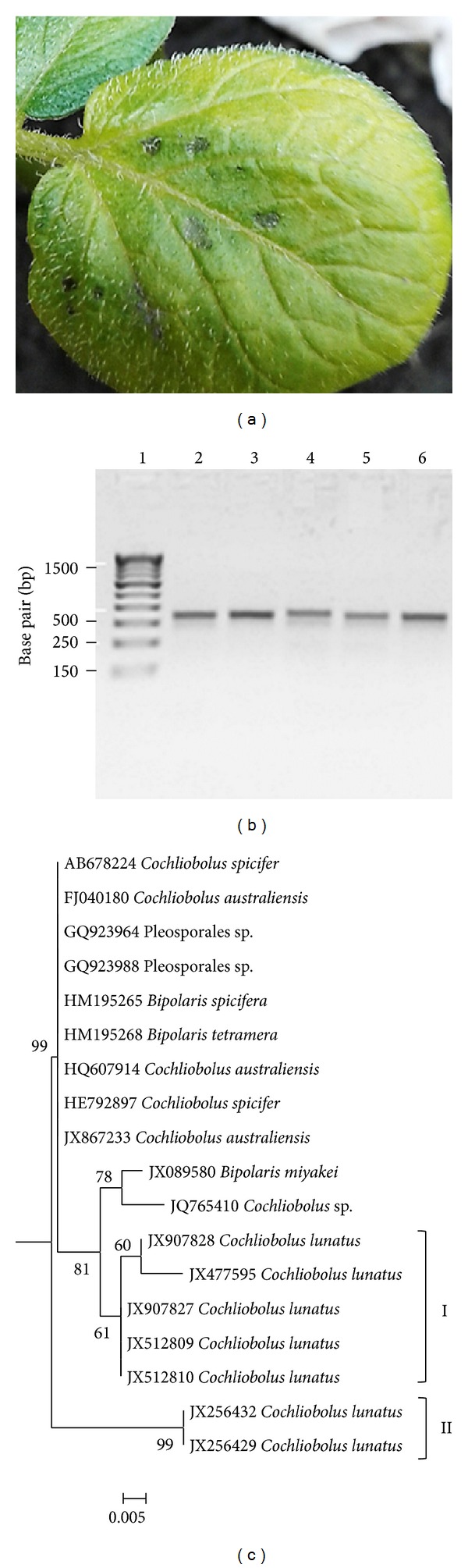
(a) Archetypal brown-to-black leaf spot disease caused by* Cochliobolus lunatus* on potato cv. Kufri Jyoti as previously validated by Koch's postulates [20]. (b) Agarose gel electrophoresis for PCR products (475 bp) from rDNA locus of* Cochliobolus lunatus* strains separated on a 2.5% agarose gel. Lane-1 DNA ladder and lane-2, -3, -4, -5, and -6 are* Cochliobolus lunatus* strains with GenBank accessions numbers JX512810, JX512809, JX907827, JX477595, and JX907828, respectively. (c) Molecular phylogenetic analysis by maximum likelihood method based on the K2 + G substitution model [21]; AIC is 871.49; BIC is 1093.89; the highest log likelihood is −429.87 and bootstrap values ≥50% from 1000 iterations are shown. Subcluster I contain strains of* Cochliobolus lunatus* which causes foliar necrosis of potato.

Taxonomic circumscription of* Cochliobolus* has undergone countless modifications in the last five decades caused by overlapping morphological characters [6, 7, 14, 15]. Furthermore, generic concepts delimiting* Bipolaris*,* Cochliobolus,* and* Pseudocochliobolus* are confused [6, 7]. Thus, ITS region of the ribosomal RNA operon was used to accurately determine the taxonomic placement of the fungi. Based on rDNA locus, we confirmed that the five fungi causing brown-to-black leaf spot disease of potato (Figures 2(a) and 2(b)) were* C. lunatus*. In the ML tree, the five strains of* C. lunatus* causing brown-to-black leaf spot disease of potato clustered (Figure 2(c), (I)), closely related to other GenBank type isolates (Figure 2(c), (II)) and distant from other* Cochliobolus* species. The nucleotide frequencies were A = 25.00%, T/U = 25.00%, C = 25.00%, and G = 25.00%. The transition-transversion bias estimated by K2 + I substitution model [21] was 2.41. The overall rate of heterogeneity between taxa was 0.01. As expected, low level single nucleotide polymorphism (SNP, 5.4%) was observed out of a total of 1188 sites at the rDNA locus. The five strains causing foliar necrosis of potato were weakly supported with bootstrap values ≤61%. As previously reported, rDNA locus do not often provide ample resolution that can allow differentiation of cryptic taxa such as* Cochliobolus* [6, 15].

**Figure 3:**
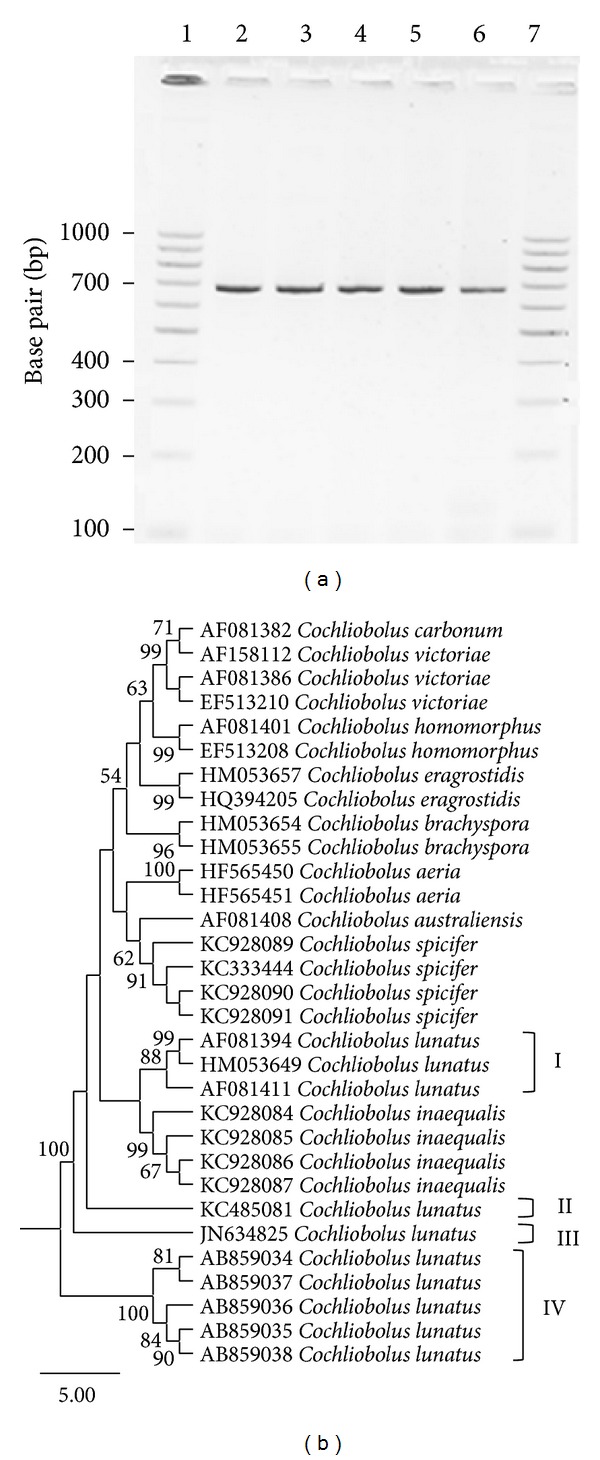
(a) Agarose gel electrophoresis for PCR products (680 bp) from* GPDH* locus of* Cochliobolus lunatus* strains separated on a 2.5% agarose gel. Lane-1 and-7 DNA ladder and lane-2, -3, -4, -5, and -6 are* Cochliobolus lunatus* strains with DDBJ accessions AB859034, AB859035, AB859036, AB859037, and AB859038, respectively. (b) Molecular phylogenetic analysis by maximum likelihood method based on the TN93 + G + I substitution model [22]; AIC is 2974.83; BIC is 3449.49; the highest log likelihood is −1494.28 and bootstrap values ≥50% from 1000 iterations are shown. Subcluster (IV) contains strains of* Cochliobolus lunatus* which causes foliar necrosis of potato.

The low bootstrap support (≤61%) generated in rDNA ML tree (Figure 2, (I)) made it difficult to determine whether the five strains of* C. lunatus* causing brown-to-black spot disease of potato in Burdwan Destrict were identical. It could be that all the strains originated from a common source but colonized in different places following dispersion. This is because* C. lunatus* abundantly produced conidia that can easily be disseminated by air to distant places. To check if the five isolates were identical or not, we used* glyceraldehyde 3-phosphate dehydrogenase* (*GPDH*) locus which had been shown to be effective in resolving* Cochliobolus* species in phylogenetic inference [6, 15]. Partial* GPDH* locus (Figure 3(a)) was sequenced, as this is one of the house-keeping genes, taken as reference in yeast and fungal systems. Based on sequence alignment for* GPDH* locus, a total of 340 SNPs out of 708 sites and 325 sites without polymorphism (45.9%) were found. Based on TN93 + G + I substitution model [22], the rate of base transition–transversion was 4.96 and the nucleotide frequencies were A = 23.73%, T/U = 18.55%, C = 33.52%, and G = 24.20% and the overall heterogeneity among taxa was 0.316. The ML tree based on* GPDH* locus discriminated the five strains of* C. lunatus* causing foliar necrosis of potato with strong bootstrap support ≥81% (Figure 3(b), (IV)). The overall mean evolutionary distance of 0.03 was observed between the five strains causing foliar necrosis of potato (Figure 3(b), (IV)) relative to other* C. lunatus* type isolates (Figure 3(b), (I), (II), and (III)).

Importantly, because the five strains of* C. lunatus* clustered based on* GPDH* locus (Figures 2(b) and 3(b)), this indicated they were closely related as also revealed on the basis of morphological descriptors (Figure 1). Additionally, bootstrap values were <100% for internal branches within the subcluster I, Figure 3(b). This indicated that the five strains which caused foliar necrosis of potato were different. Although the five* C. lunatus* strains might have adapted in potato for their nutritional requirements in the same geoclimatic zone, it was not possible to determine their origin. Importantly, it is shown that pathogenic fungi are capable of adapting to the genetic background of their host, thus forming new physiological and virulent races [26]. This is generally a slow progressive process determined mainly by the degree of the pathogen-host specific interactions [27]. Collectively, because of some phenotypical variations such as colonies growth pattern, presence or absence of stromatic tissues, colours of conidia and colonies (Figure 1), and strong bootstrap support (>81%) for clustered and unclustered strains (Figure 3(b), (I), (II), and (III)),* C. lunatus* strains have evolved divergently.

### 3.2. Host-Range Diversity

Herein, the term host-range diversity described the group of different hosts on which* C. lunatus* successfully thrived on such as monocots, dicots, invertebrates, and vertebrates. The known hosts of* C. lunatus* presented by Sivanesan [14] in 1987 are plant species, namely,* Cynodon* sp.,* Oryza* sp.,* Pennisetum* sp.*, Saccharum* sp.,* Sorghum* sp.,* Triticum* sp., and* Zea* sp. There was no up-to-date account on the new host gained by* C. lunatus* since Sivanesan [14] account. By exploring the public repositories, we found that* C. lunatus* have gained hosts within host groups such as monocots, dicots, vertebrates, and invertebrates in the last decade (Table 1). New hosts gained in the last decade are* Homo sapiens*,* Musa acuminata*,* Jatropha curcas*,* Echinochloa* sp.,* Arecales* sp.,* Cyperaceae* sp.,* Panicum* sp.,* Setaria italic*,* Solanum tuberosum* L.,* Glycine max* L.,* Nelumbo nucifera*,* Eugenia jambolana*,* Actinidia deliciosa*,* Actinidia* sp.,* Trachymyrmex septentrionalis,* and* Cyphomyrmex wheeleri* (Table 1), geographically distributed across Africa, Asia, North America, South America, and Europe. It is worth noting that Europe was not included in Sivanesan [14] report by 1987 (http://www.tifton.uga.edu/fat/disfunt2.htm). Other* C. lunatus* new hosts reported [19, 28–40] in the last decade without nucleotide sequence information are depicted (Table 2).

This study seeks to advance insights on the host-range diversity allowing the dynamic movement of* C. lunatus* observed in the last decade (Tables 1 and 2). From Table 1, it is understood that* C. lunatus* exploit two kingdoms, notably plant and animal, switching among monocots, dicots, invertebrates, and vertebrates. The paradigm-shift from a plant colonizer sensu stricto to a vertebrate and invertebrate invader (Tables 1 and 2) indicates that* C. lunatus* have acquired special strategies to switch hosts. The question arises as to why* C. lunatus* display extensive host-range diversity in a given biota.

Although studies have shed light on specific aspects of* C. lunatus* pathogenicity such as induce-virulence variations on maize crop [41], virulence differentiation on maize crop [42], secretome weaponries on potato crop [43], and heat-dependent virulence on* Lolium* spp. [44], the nutritional evolution of* C. lunatus* is unresolved. Intriguingly, host shifting dynamics is not well understood and it has been argued that (1) close proximity to host is prerequisite for pathogens to jump from a natural host to a new host [45], (2) the future host must act as the substrate [15, 45], and (3) compatible factors promoting infection must be present [45, 46]. Importantly, most host-switching pathogens self-protect themselves by producing high level pigment such as melanin to deal with the host defense [42, 47, 48]. Additionally,* C. lunatus* profusely produced melanized colonizing hyphae during invasion in potato [43] and non-host-specific toxin such as methyl-5-[hydroxymethylfuran-2-carboxylate] in maize [49], to suppress the host defense.

Nonetheless, the above-mentioned factors seem more likely to be limited in explaining how* C. lunatus* gain access to hosts and not how* C. lunatus* spreads in a given biota and prevails as an environmental hazard.* C. lunatus* had extensively been used as mycoherbicide formulations in the past decade [8–13]. Remarkably, Zhang et al. [12] fused the protoplast of* Helminthosporium gramineum* and* C. lunatus*, to generate a strain with high potential to produce conidia, phytotoxin ophiobolin, and improved potential to control rice weed. Introduction of genetically manipulated strains and unmodified strains of* C. lunatus* could have hazardous implications to the environment. This is because, in some cases,* C. lunatus* failed to control the targeted weeds but caused severe damages in economically important crops in the same biota. For instance,* C. lunatus* isolated from barnyardgrass and used as mycoherbicide failed to control competitive weeds in rice fields but severely damaged bean varieties [8]. Nevertheless, the effectiveness of a biocontrol in the fields depends on the environmental conditions of a given biota, especially humidity and temperature [10].* C. lunatus* exhibits a temperature-dependent virulence [43, 44] and its introduction into the environment without a precise prediction of the geoclimatic conditions, that is, humidity and temperature, can prove harmful; consequently, it disequilibrates the interaction dynamics of the organisms dwelling in the same biota.

Owing to the divergence in evolution (Figure 1, (I) and (II)) and the emergence of unclustered strains (Figure 2, (I) and (II)), it is clear that strains of* C. lunatus* have coevolved with their different hosts translated by their different placement in ML tree inference and speciation in their nutritional requirements (Tables 1 and 2). In keeping with the results of the evolutionary disparity, the global control of* C. lunatus* diseases would require tremendous exertion. This is because in an intermixed network of host-groups,* C. lunatus* strains from different hosts, genetically distant ((IV) versus (I), (II), and (III), Figure 2(b)) and found in the same geographic zone, would readily invade putative hosts regardless of their temporal host-groups. For instance in the last three years in India,* C. lunatus* have invaded strawberry [19],* Mimusops elengi* [37],* Amaranthus spinosus* [36],* Grewia optiva* [33],* Clerodendrum indicum* [40], and potato [20, 48]. Noteworthy, these hosts were spatially and temporally distant. With this illustration, it is clear that the host-pathogen proximity hypothesis and host relatedness hypothesis, where a given pathogen switches to new species closely related to the original host, might all apply for* C. lunatus*.

## 4. Conclusions

From an evolutionary viewpoint, the variations observed in* C. lunatus* colony, conidia size, conidia colour, conidia texture, and the presence or absence of stromata should be regarded as prominent acquired adaptational traits. These characteristic traits were not consistent between the five strains causing foliar necrosis of potato but provided indicators for generic circumscription. Phenotypic intraspecific variations can obscure placement of* Cochliobolus* species and make correlation to phylogeny difficult. As shown,* C. lunatus* have considerable ecological and economic importance being a highly successful colonizer in monocots, dicots, vertebrates, and invertebrates. On this basis, the purpose as a biocontrol agent is overshadowed by its virulent and indiscriminate destructive potential in the ecosystem. For this reason, we suggest that the use of* C. lunatus* as mycoherbicide should be stopped.

## Figures and Tables

**Table 1:** Information associated with rDNA locus of *C. lunatus* deposited in the GenBank, DDBJ, and EMBL in the last decade on different host groups such as invertebrates, vertebrates, monocots, and dicots from Asia, Africa, South America, North America, and Europe.

Accessions	Host	Host group	Geographic origin	Date of report
EU828350	Allelopathic rice (leaf)	Monocots	China (Fujian)	16-Jul-2008
GQ179977	*Musa acuminata *		China	27-Jun-2009
GQ328852	*Zea* sp. (seed)		USA (Peoria)	25-Jun-2009
JF798505	*Jatropha curcas *		Mexico	16-Feb-2012
JX256435	*Oryza *sp. (leaf)		Thailand	10-Sep-2012
HQ248192	*Arecales* (Oil palm, leaf)		Colombia	31-Oct-2010
AF163082	*Oryza *sp.		China (Hong Kong)	27-Jul-2000
GQ328851	*Zea* sp. (seed)		US (Peoria)	05-Aug-2009
FJ040177	*Oryza *sp. (grains)		China (Zhejiang)	20-Sep-2008
EF189917	*Echinochloa *sp. (leaf)		China (Zhejiang)	22-Jan-2007
JN207244	*Cyperaceae* sp. (Sedges, leaf)		Venezuela (Northwest)	22-Jun-2012
JX256436	*Echinochloa *sp. (leaf)		Thailand	10-Sep-2012
JX256432	*Panicum *sp.		Thailand	10-Sep-2012
JX256444	*Panicum *sp.		Solomon Island	10-Sep-2012
HQ130484	*Panicum virgatum* (switchgrass)		USA (Tennessee)	29-Aug-2012
JN943425	*Echinochloa *sp.		Japan (Kochi)	21-Dec-2011
JN943426	*Setaria italica* (leaf)		Japan (Kagoshima)	21-Dec-2011
JN943424	*Setaria italica* (leaf)		Japan	21-Dec-2011

JX512810*	*S. tuberosum* L. (leaf)	Dicots	India (Burdwan)	20-Aug-2012
JX512809*	*S. tuberosum* L. (leaf)		India (Burdwan)	20-Aug-2012
JX907827*	*S. tuberosum* L. (leaf)		India (Burdwan)	09-Sep-2012
JQ936200	*Glycine max *L. (leaf)		Brazil	16-Apr-2012
JX477595*	*S. tuberosum* L. (leaf)		India (Burdwan)	12-Aug-2012
JX907828*	*S. tuberosum* L. (leaf)		India (Burdwan)	09-Sep-2012
JQ701798	*Nelumbo nucifera* (leaf)		China (Jiangxi)	01-Jul-2012
JQ765410	*Ipomoea carnea* (leaf)		India	03-Jul-2012
KC937052	*S. tuberosum* L. (leaf)		India	12-Aug-2013
KF031026	*Eugenia jambolana *		India	11-May-13
JX256445	*Actinidia deliciosa *		Solomon Island	10-Sep-2012

JF819163	*Actiniaria* sp.	Invertebrates	China (Yushan)	19-Apr-2011
JQ717321	Corales		China (Guangdong)	13-Aug-012
HQ608077	*Trachymyrmex septentrionalis *		USA (Texas)	15-Nov-2011
HQ608020	*Cyphomyrmex wheeleri *		Brazil	15-Nov-2011
JQ388928	M*arine sponge *		Panama	01-Jun-2012
HQ607975	*Cyphomyrmex wheeleri *		USA (Texas)	15-Nov-2011

JX256429	Human lungs biopsy	Vertebrate	USA	10-Sep-2012
HE861835	Human nasal nostrils		Spain	23-Jul-2013
KC288118	Human subcutaneous tissue		Brazil	21-Nov-2012

EU759980	Plant	Unknown	Egypt	25-Jun-2008
HQ174562	Unknown		China (Shandong)	22-Feb-2011
JN943422	Unknown		United kingdom	17-Apr-2012
FJ792584	Medicinal plants		China (Jiangsu)	30-Mar-2009
AF071339	Unknown		Canada	10-Jun-1998

JX077054	Wetland sediment	Soil	China (Zhejiang)	17-Jul-2012

*Accessions corresponding to isolates causing brown-to-black leaf spot disease of potato reported in this study.

**Table 2:** New *Cochliobolus lunatus* hosts reported in the last decade without sequence information.

Host origin	Geographic origin	Year of report	Reference
*Dioscorea* sp.	Nigeria	2005	Amusa et al. [28]
*Chrysalidocarpus lutescens *	New Zealand	2006	Braithwaite et al. [29]
*Saccharum officinarum *	Japan	2008	Nishi et al. [30]
*Passiflora edulis* f. flavicarpa Deg.	Philippines	2009	Marvin and Naomi [31]
*Pennisetum typhoides *	Pakistan	unknown	Azhar et al. [32]
*Fragaria* × *ananassa* Dutch (Strawberry)	India	2010	Verma et al. [19]
*Grewia optiva *	India	2011	Cvetomir [33]
*Basella rubra *	India	2011	Pandey et al. [34]
*Mimusops elengi* Linn	India	2011	Selima et al. [35]
*Amaranthus spinosus *	India	2011	Sharma et al. [36]
*Vicia faba *	Egypt	2012	Saleem et al. [37]
*Allium sativum* L.	India	2013	Ghangaonkar [38]
Lake water (Fishes)	India	2013	Pratibha et al. [39]
*Clerodendrum indicum *	India	2013	Mukherjee et al. [40]
